# The potential role of epigenetic modulations in BPPV maneuver exercises

**DOI:** 10.18632/oncotarget.9446

**Published:** 2016-05-18

**Authors:** Kun-Ling Tsai, Chia-To Wang, Chia-Hua Kuo, Yuan-Yang Cheng, Hsin-I Ma, Ching-Hsia Hung, Yi-Ju Tsai, Chung-Lan Kao

**Affiliations:** ^1^ Department of Physical Therapy, College of Medicine, National Cheng Kung University, Tainan, Taiwan; ^2^ Department of Physical Medicine and Rehabilitation, Taipei Veterans General Hospital, Taipei, Taiwan; ^3^ Institute of Clinical Medicine, School of Medicine, National Yang-Ming University, Taipei, Taiwan; ^4^ Department of Sports Sciences, University of Taipei, Taipei, Taiwan; ^5^ Department of Physical Medicine and Rehabilitation, Taichung Veterans General Hospital, Taichung, Taiwan; ^6^ Department of Neurological Surgery, Tri-Service General Hospital, National Defense Medical Center, Taipei, Taiwan; ^7^ Department of Physical Medicine and Rehabilitation, School of Medicine National Yang-Ming University, Taipei, Taiwan

**Keywords:** benign paroxysmal positional vertigo, microRNA, Sirtuin 1, maneuver exercises, Gerotarget

## Abstract

Benign paroxysmal positional vertigo (BPPV) is one of the most common complaints encountered in clinics and is strongly correlated with advanced age or, possibly, degeneration. Redistribution exercises are the most effective approaches to treat BPPV, and canalith repositioning procedure (CRP) cure most BPPV cases. However, the mechanisms through which the treatment modulates systemic molecules in BPPV patients remain largely unknown. In this study, we report that the miR-34a and Sirtuin 1 (SIRT1) genes correlated with the treatment effects of CRP in BPPV subjects. We found that miR-34a expression was largely inhibited and SIRT1 expression was significantly reversed after BPPV maneuver treatment. We also confirmed that the PPAR-γ, PGC-1 and FoxO gene expressions were decreased immediately after canalith repositioning procedure (CRP) for BPPV, and were largely increased after a complete cure of BPPV. Moreover, we observed that after a complete recovery of BPPV, the ROS concentrations, pro-inflammatory cytokine concentrations and p53 expression levels were attenuated. We conclude that BPPV treatment might involve some epigenetic regulations through the mediation of miR-34a, SIRT1 functions and repression of redox status.

## INTRODUCTION

Benign paroxysmal positional vertigo (BPPV) is one of the most common complaints encountered in otology, neurology and rehabilitation clinics. The typical presentation of BPPV is intense, brief vertigo spells that are provoked by a change in head position [1]. Benign paroxysmal positional vertigo is believed to be the consequence of displaced otoconia within the semicircular canals located in the inner ear [2]. Individuals with a history of head trauma or ear infections are prone to developing BPPV [3]. Moreover, BPPV occurrence is highly related to advanced age or, possibly, degeneration. One previous study suggested that age, migraines, hypertension, hyperlipidemia and stroke are independently associated with BPPV [4]. The occurrence and recurrence rates are increased among the senior population [5].

Repositioning maneuvers are effective interventions for BPPV. Brandt and Daroff designed the first operative physical therapy of redistribution exercise [6]. The development of redistribution exercise was based on the theory of cupulolithiasis, which explains the mechanism of BPPV as calcium deposits adhering to cupula. The Brand-Daroff redistribution maneuver exercise cured approximately 92.68% of BPPV patients, with a recurrence rate of approximately 4.22% [7]. Another maneuver exercise, modified by Dr. Epley et al., the so-called canalith repositioning procedure (CRP), has been extensively used for the treatment of posterior canal BPPV [8]. The canalith repositioning procedure is based on the canalolithiasis theory, which indicates that BPPV is caused by free-floating debris in the ampullofugal branches of semicircular canals. The free-floating debris moves to the most dependent part of the semicircular canals relative to gravity during head movements, causing transient spells of vertiginous episodes [1].

With the advancement of biomolecular medicine, disease etiologies can be investigated more thoroughly by exploring the underlying cellular pathways. microRNAs (miRNAs) have been found circulating in human blood, particularly in cell-free plasma. miRNAs play vital roles in the regulation of pathological communications. Importantly, in human blood, circulating plasma miRNAs appear to be secreted from the cellular compartment [9]. miR-34a, one well-known miRNA involved in cell cycle regulation and p53-related apoptosis signaling transduction, was first revealed as a posttranscriptional regulator of SIRT1 [10]. Silencing of miR-34a function by antisense oligonucleotides attenuates p53 acetylation and PUMA expression at the protein level [11]. miR-34a mediates human endothelial cell pro-senescence responses, increases the fraction of cells in G1 phase of the cell cycle and impedes angiogenesis by modulating SIRT1 and forkhead box O transcription factor 1 (FoxO1) [12]. Shephali et al. revealed that miR-34a expression is enriched in circulating blood plasma in Alzheimer's disease [13]. Julien et al. also demonstrated that SIRT1 reduction parallels the accumulation of tau in Alzheimer's disease, suggesting that miR-34a and SIRT1 play important roles in the progression of neurodegenerative diseases.

Vast amounts of literature have indicated that BPPV increases with advanced age. Recent knowledge implied BPPV might be a results of otconia degeneration [14]. However, the mechanisms behind this degeneration is still unclear. Pro-inflammatory regulators are activated during the aging process. NF-κB is one of the most widely known mediators controlling pro-inflammatory signaling [15]. Chronic inflammation occurs in the brain of the senescence-accelerated (SAMP8) mouse, and it constitutively activates pro-inflammatory genes through NF-κB up-regulation [16]. Cyclooxygenase-2 (COX-2) is an important enzyme in the prostaglandin (PG) synthetic cascade during the aging process [17]. Prostaglandin E2 (PGE2) secretion was found to be up-regulated in aged mice (23 months old) only, which provides some knowledge regarding the senescence-related pro-inflammatory process [18]. It has also been shown that signaling transduction *via* the FoxO longevity factors and SIRT1 can functionally mitigate the NF-κB pathway and simultaneously repress the inflammation-aging process [19]. Currently, repositioning maneuver exercises are believed to be the only effective non-pharmacological interventions that can cure BPPV through mechanical ways [20]. The ultimate goal of these maneuvers is to reposition the otoconia into the cupula. Whether these maneuvers involve biomolecular mechanisms, such as inflammation and senescence, has not yet been reported.

The primary aim of this study was to investigate the epigenetic regulation of miR-34a expression in circulating blood samples after CRP in patients with the canalolithiasis type of posterior canal BPPV. Because the SIRT1 gene is highly related to aging and neurodegenerative diseases [21], we also hypothesize that the expression of the SIRT1 gene, which is directly regulated by miR-34a, along with the SIRT1 downstream genes would be positively activated after complete treatment for BPPV.

## RESULTS

### Subject characteristics

A total of 20 BPPV patients (mean age = 62.8 ± 14.4; M:F = 3:17; left posterior canal BPPV = 5, right posterior canal BPPV = 15) completed treatment and blood sampling for this study. The duration for complete resolution of BPPV was 8.53 ± 5.27 days. During the follow up visits, all patients had symptomatic relief and were free from positional vertigos. Using infra-red video goggles, no nystagmus was observed in Dix-Hallpike positions.

### Patient miR-34a and SIRT1 expression levels were altered upon completion of BPPV treatment

**Figure 1:**
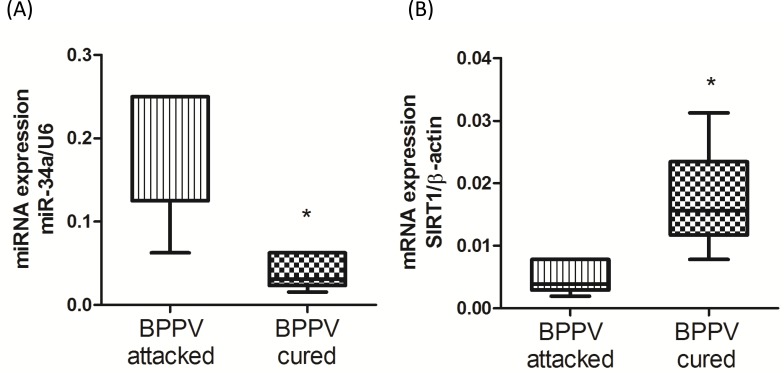
Expression levels of **A.** miR-34a and **B.** SIRT1 in BPPV-attacked and BPPV-cured patients after treatment of maneuver exercise. Total blood mRNAs were isolated from patients, the miR-34a and SIRT1 expression were investigated after maneuver exercise intervention using real-time PCR.

miR-34a plays an important role in the regulation of neurodegenerative diseases [13]. Moreover, Li's work confirmed concordant increases in miR-34a expression in the brain and plasma. Li et al. concluded that circulating miR34a was an RNA based, noninvasive biomarker for brain aging [10]. Our previous publication reported that BPPV was positively related to aging [5]. Therefore, we investigated miR-34a levels in plasma samples using quantitative reverse transcription PCR (qPCR) analyses. As shown in Figure 1A, miR-34a expression was relatively higher in the blood samples of the BPPV attacked patients. However, miR-34a expression was significantly reduced after CRP treatment. SIRT1 is an important protective factor against neurodegenerative diseases in humans. A decrease in SIRT1 function has been reported to occur with increasing age [22]. Figure 1B shows that blood SIRT1 expression levels were relatively repressed in BPPV samples; however, the expression pattern was reversed following a BPPV cure (*P* < 0.05).

### DBC1 expression level was affected in clinical treatment

**Figure 2:**
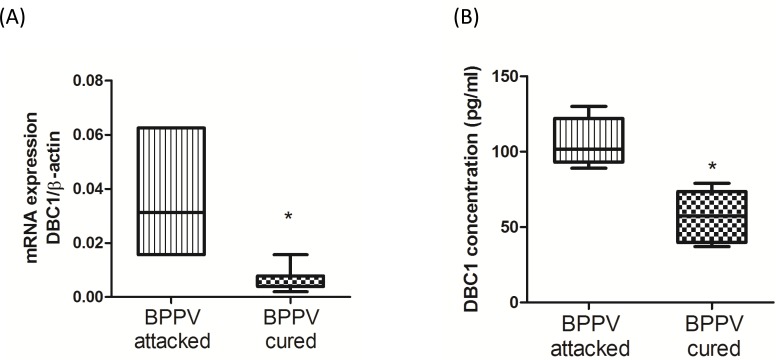
Expression levels of DBC1 in BPPV-attacked and BPPV-cured patients after treatment of maneuver exercise **A.** Total blood mRNAs from BPPV-attacked and BPPV-cured patients were isolated from patients, and the DBC1expression was tested after maneuver exercise intervention using real-time PCR. **B.** Blood plasma was isolated from patients. DBC1 concentration was investigated using ELISA kits.

Deleted in breast cancer-1 (DBC1) was firstly described from the observation that DBC1 is deleted in some human breast cancers [23]. DBC1 directly interacted with the catalytic domain of SIRT1, thereby inhibiting SIRT1 activities [24]. Moreover, DBC1 is thought to be one key co-factor for the mediation of the IKK-β-NF-κB signaling pathway [25]. DBC1/SIRT1 complex may act an important role in regulation of aging and inflammatory responses. Figure 2A and 2B shows DBC1 mRNA and protein expression levels were attenuated following a BPPV cure (*P* < 0.05).

### Following the canalith repositioning procedure, p53, PGC-1 and PPAR-γ expression levels were changed

**Figure 3:**
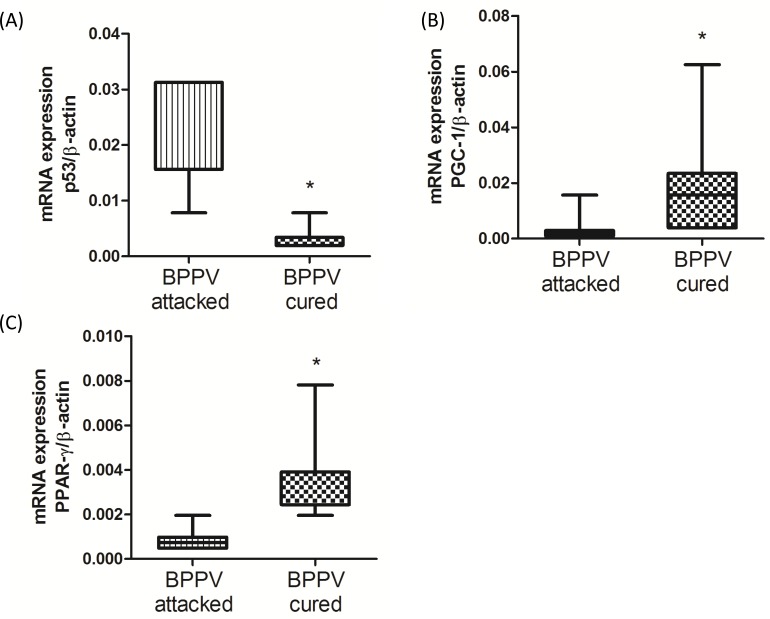
Expression levels of SIRT1 downstream genes in BPPV-attacked and BPPV-cured patients after treatment of maneuver exercise Total blood mRNAs were isolated from patients, and the **A.** p54, **B.** PPAR-γ and **C.** PGC-1 expression were tested before and after maneuver exercise intervention using real-time PCR.

miR-34a functions in the SIRT1-mediated deacetylation of p53 and in the neuronal-protective signaling pathway [26]. Moreover, SIRT1/p53/miR-34a is involved in a positive feedback loop involved in the regulation of miR-34a expression. Indeed, up-regulated miR-34a mitigates SIRT1 function, and attenuates p53 deacetylation, leading to an increase in acetylated p53 transcriptional activity [11]. We previously suggested that miR-34a expression were reduced and the SIRT1 expression level were up-regulated after clinical BPPV rehabilitation. We turned our attention on investigating the p53 level in blood samples of BPPV patients immediately after (BPPV attacked) and 1-2 weeks after (BPPV cured) CRP treatment. As shown in Figure 3A, using real-time PCR, we confirmed that p53 mRNA levels were significantly higher in the BPPV samples compared with the BPPV-cured samples (*P* < 0.05). PPAR-γ coactivator-1α (PGC-1α) and peroxisome proliferator-activated receptor-gamma (PPAR-γ) are important SIRT1 downstream target genes [27]. We used real-time PCR to further confirm whether the gene expression of PGC-1α and PPAR-γ was involved in BPPV episodes. The data shown in Figure 3B and 3C suggested that the blood expression levels of PPAR-γ and PGC-1 were significantly lower in BPPV attacked patients compared with the BPPV cured (*P* < 0.05). Our results indicated that after maneuver intervention, patients’ biological and physiological functions involving the repression of miR-34a expression and the activation of SIRT1, PGC-1 and PPAR-γ were enhanced.

### The expression of FoxO family genes was mitigated during BPPV episodes

**Figure 4:**
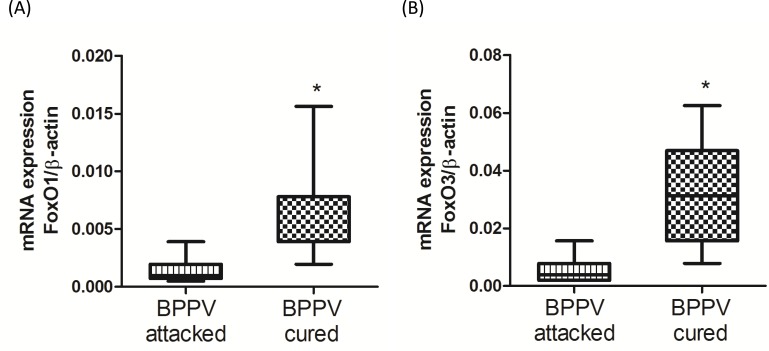
Expression levels of FoxOs genes in BPPV-attacked and BPPV-cured patients after treatment of maneuver exercise Total blood mRNAs were isolated, and the **A.** FoxO1 and **B.** FoxO3 expression were observed before and after maneuver exercise intervention using real-time PCR.

It has been reported that the FoxO family of genes modulate a direct effect on hundreds of coupled side- and downstream genes [28]. FoxO genes are strongly associated with human longevity, aging and degeneration [29]. FoxO genes are important SIRT1 downstream targeting in regulation of cellular function by deacetylation of FoxO genes [30, 31]. We were interested to observe the expression levels of FoxOs in patients during BPPV episodes. As shown in Figure 4A and 4B, we found that FoxO1 and FoxO3 expression was significantly mitigated in BPPV attacked samples compared with BPPV cured samples (*P* < 0.05).

### The expression of FoxO family genes was mitigated during BPPV episodes

**Figure 5:**
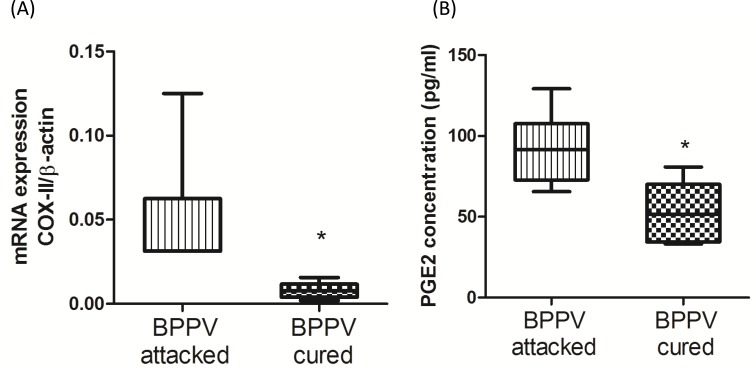
Pro-inflammatory evens in BPPV-attacked and BPPV-cured patients after treatment of maneuver exercise Total blood mRNAs were isolated, and the **A.** COX-II expression were observed before and after maneuver exercise intervention using real-time PCR Blood plasma was isolated from patients. **B.** PGE2 concentration werewere investigated before and after exercise intervention using ELISA kits.

**Figure 6:**
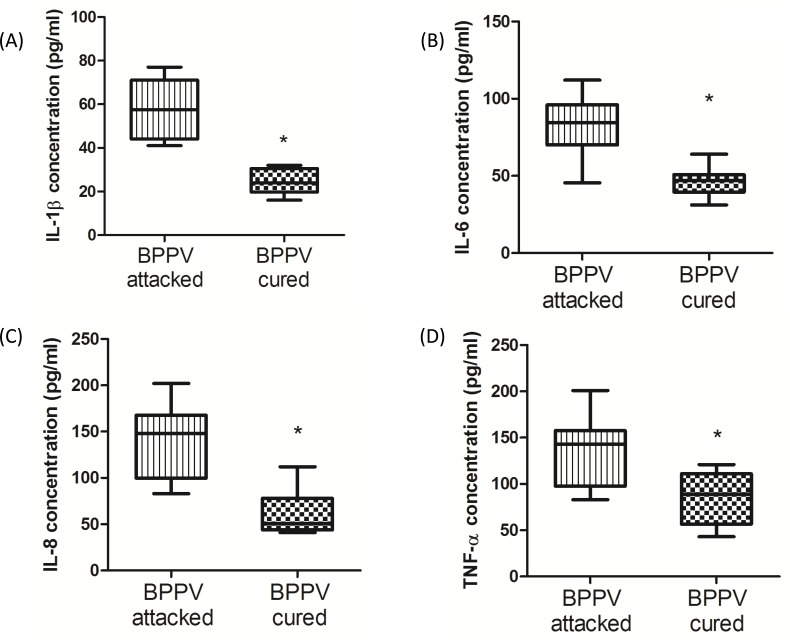
Pro-inflammatory cytokines concentration in BPPV-attacked and BPPV-cured patients after treatment of maneuver exercise Blood plasma was isolated from patients. **A.** IL-1β, **B.** IL-6, **C.** IL-8 and **D.** TNF-α were investigated before and after exercise intervention using ELISA kits.

During the aging process, genes involved in inflammatory responses are elevated in human brains [32]. Glass et al. suggested that the increase in SIRT1 expression inhibits pro-inflammatory responses, thus highlighting the potent anti-aging effects of neuronal degenerative diseases [33]. Nuclear factor kappa B (NF-κB) is a key regulator of inflammatory pathways [34]. The NF-κB binding domain has been identified as the motif most highly involved with the aging process by bioinformatics analyses [1]. Furthermore, COX-2 expression is known to be enriched during senescence, leading to an up-regulation of prostaglandin E2 (PGE2) production [35]. Activated NF-κB binds to COX-2 promoter region is necessary to facilitate COX-2 activation [36]. On investigating the levels of COX-2 and PGE2 in BPPV patient blood samples, we found that COX-2 mRNA expression was higher in the BPPV attacked samples than the BPPV cured samples (Figure 5A). We also confirmed that PGE2 concentrations were higher in the BPPV attacked samples than in the BPPV cured samples (Figure 5B). SIRT1 is considered as an important NF-κB inhibition, hence, the inhibitory ability of SIRT1 in anti-inflammation and reduction of pro-inflammatory responses [37] [38]. In Figure 6, we showed that IL-1, IL-6, IL-8 and NF-κB concentrations were significantly higher in the BPPV attacked samples by ELISA assays.

### Oxidative stress is increased in BPPV patients during BPPV episodes

**Figure 7:**
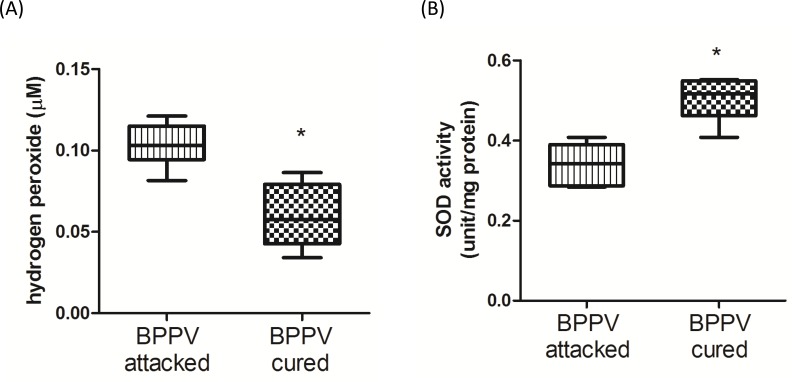
Oxidative status in BPPV-attacked and BPPV-cured patients after treatment of maneuver exercise Plasma was isolated from whole blood and **A.** H2O2 concentration and **B.** SOD activity were investigated using kits.

Reactive oxygen species (ROS) have been shown to be a major factor involved in the progression of neurodegenerative diseases [39]. An increase in lipid peroxidation and a decrease in polyunsaturated fatty acids have been confirmed in neurodegenerative diseases [40]. Mir-34a and SIRT1 have been shown to functionally regulate redox status. For example, mir-34a modulates cell apoptosis by regulating ROS production and NOX2 expression [41], and SIRT1 inhibits ROS formation *via* mediating mitochondrial biogenesis [42]. We confirmed that hydrogen peroxide concentrations were relatively higher in BPPV- attacked samples than in BPPV-cured samples (Figure 7A). Benzi et al. reported that age-related changes in antioxidant function are involved in enzymatic and nonenzymatic events [43]. In Figure 7B, we shown the SOD activities were significantly reduced in patients with BPPV after treatment.

## DISCUSSION

**Figure 8:**
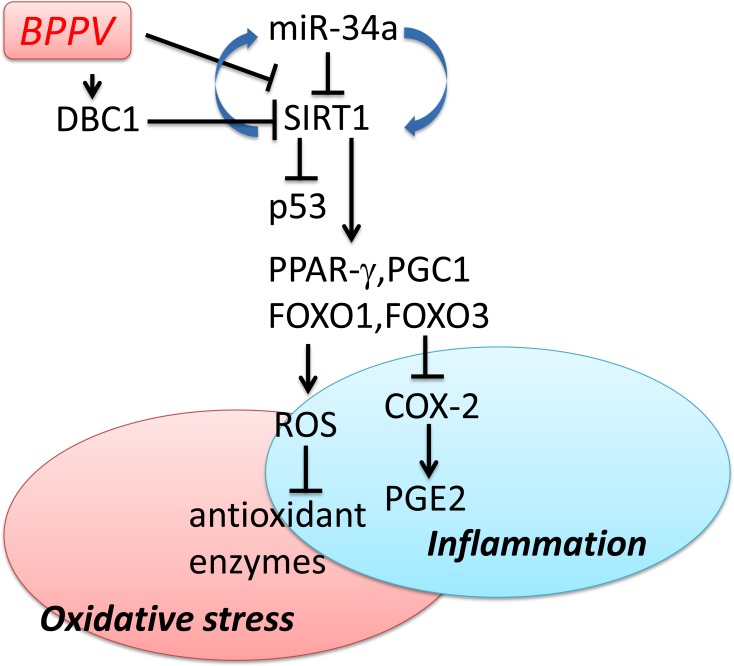
Schematic diagram showing the possible epigenetic regulation of BPPV by maneuver exercise As depicted, maneuver exercise intervention reduces miR-34a and enhances SIRT1 expression and also up-regulates PPAR-γ, PGC-1 expression. Maneuver exercise intervention improves antioxidant enzyme activity and reduces the ROS concentration in blood. The → indicates activation or induction, and ┤indicates inhibition or blockade.

To date, the recommended treatment for BPPV remains to be the repositioning maneuvers/procedures [44]. The dislodged calcium carbonate crystals, which presumably arise from within the semicircular canals, can be relocated back into the vestibule through this non-invasive CRP maneuver [45]. In our study, we demonstrated miR-34a levels were significantly higher and SIRT1 expression levels were significantly lower in blood samples of BPPV patients immediately after BPPV treatment (BPPV attacked) compared with the samples collected after completion of treatments. We also suggested that DBC1 is affected after the repositioning treatments of BPPV. Moreover, FoxO, PGC-1α, PPAR-γ and p53 expression levels were also altered after recovery of BPPV. We proposed the hypothesis that following successful maneuver exercises, pro-inflammatory responses in the blood were corrected, and the oxidative stress in BPPV subjects were thus attenuated (Figure 8).

BPPV occurs with advancing age, and more frequently in females. Therefore, the etiology of BPPV may be partly due to degeneration [46]. The peak incidence of BPPV occurs in individuals between 50 and 70 years of age [4]. In patients > 70 years of age, the occurrence and recurrence rates are significantly higher [5]. miRNAs in human blood samples have been considered a key class of biomarkers, as they have been shown to influence physiological conditions ranging from cancer to brain dysfunction. The level of circulating miR-34a in human plasma has been linked to Alzheimer's disease [13]. Increased miR-34a levels were also found in aged mouse brains [10]. In this study, we demonstrated for the first time that miR-34a expression levels were higher in the BPPV attacked samples; however, whether BPPV episodes are directly related to degeneration requires further investigation. The contribution of the miR-34a/SIRT1 axis to senescence has been validated that miR-34a functions to induce endothelial senescence *via* SIRT1 regulation [48]. SIRT1 has its protective roles in different neurodegenerative diseases, such as Alzheimer's disease and Parkinson's disease [47]. It has been well documented that one of the SIRT1 downstream gene, DBC1, is a main negative regulator of SIRT1 [24]. In addition, DBC1 is associated with IKK-β to increase NF-κB transcriptional activities [25]. Calliari et al reported SIRT1 activation mitigates neurodegeneration through DBC1inhibition [48]. These finding suggest that DBC1 might play a key role in the mediation of neurodegenerative diseases. BPPV has been considered as a degenerative disease [49] and our study echoes that DBC1 mRNA and protein expression levels were attenuated following a BPPV cure.

A previous study indicated that collapse of mitochondrial activities may lead to the formation of neurodegenerative diseases [50]. One previous literature found that SIRT1 could activate PGC-1 *via* deacetylation in the mitochondria, thereby controlling mitochondrial biogenesis and oxidative phosphorylation [51]. PGC-1 activation is important for the facilitation of reactive oxygen species (ROS)-detoxifying enzymes. PGC-1 dysfunction activates oxidative stress and neurodegenerative disease [52]. In this study, we confirmed that blood PGC-1 and ROS levels can be reduced after maneuver treatment in BPPV patients (Figure 3B and Figure 7A), indicating that mitochondrial mediated oxidation changes may be involved in the recovery of BPPV. The FoxO family has been correlated with aging and neuronal degenerative damage through modulation of SIRT1.FOXO up-regulates SOD and catalase, which delays axonal degeneration and neuronal loss [53]. Pino et al. also suggested that FoxO3 determines the accumulation of α-synuclein and mediates the fate of dopaminergic neurons in the substantia nigra [54]. Taken together, the activation of FoxO family genes may be a novel tactic in the intervention of diseases related to degeneration, possibly in diseases like BPPV (In Figure 4).

BPPV is the most common form of vestibular dysfunction in the elderly [55]. This mechanism might involve the up-regulation of redox-sensitive transcription factors *via* the increasing effects of inflammatory injuries during senescence [56]. Goto et al suggested that BPPV is correlated to oxidative stress and pro-inflammatory responses [57].SIRT1 can directly interact with p65 [58] and mitigate the transcription activity of activator protein-1 (AP-1), which inhibits the important pro-inflammatory gene cyclooxygenase-2 (COX-2) [59]. In Figure 5 and Figure 6, we revealed that COX-2 expression levels as well as pro-inflammatory cytokines PEG2, IL-1, IL-6, IL-8 and TNF-α were significantly higher during BPPV attacks. These finding suggest that inflammation may be persistently activated in BPPV episodes. Previous bioinformatics findings revealed that miR-34a has many potential target genes, and cellular investigations have identified several of these targets, such as SIRT1 [11, 60]. Yamakuchi et al. reported that miR-34a targets the 3′UTR of SIRT1. The same report indicated that miR-34a inhibition reduces SIRT1 expression, thereby regulating cell apoptosis [11]. Interestingly, SIRT1 overexpression protected against pre-miR-34a-induced senescence in endothelial cells, indicating that miR-34a contributes to cellular senescence through the repression of SIRT1 function [12].

One limitation of our study is its cross-sectional design. A longitudinal study will be valuable to determine the roles of miR-34a/SIRT1 in long-term effects of CRP. Second, only blood and plasma samples were tested in this study, and therefore, our findings may not be able to reflect the exact physiological processes in human. However, our data has shed a light on the biomolecular changes in rehabilitation treatment of BPPV. Further parameters and tools are required in our future studies to further elucidate the pathophysiological conditions in CRP treatment. Moreover, we previously reported that the plasma of post-treatment BPPV patients had lower mean MDA levels than pretreatment samples. We also found that the mean levels of the antioxidative enzyme SOD were higher in plasma from post-treatment BPPV patients than their pretreatment samples. Notably, oxidative stress remained higher in BPPV post-treatment samples than samples from non-dizzy subjects despite the fact that the reduction in oxidative stress paralleled symptomatic relief in BPPV patients after CRP [61]. The expression levels of miR-34a and SIRT1 could also be compared between patients and healthy individuals to further validate the hypothesis that BPPV treatment might involve some epigenetic regulations through the mediation of miR-34a, SIRT1 functions and repression of redox status.

## MATERIALS AND METHODS

### BPPV diagnosis and treatment

Patients with chief complaints of positional vertigo were examined using the Dix-Hallpike test (sensitivity 82%, specificity 71%) [62, 63]. The site of semicircular canal involvement was determined by the direction of nystagmus observed in the infrared video fixation-block goggles (Micromedical Computerized Real Eyes infrared video Frenzel with single, pivotal camera, Micromedical Technologies, Illinois, USA). Patients with up and torsional beat nystagmus that lasted for less than 1 minute on the left or right side of the Dix-Hallpike position were included in this study. Therefore, only patients with posterior canal canalolithiasis BPPV [1] were included in this study. Patients were then treated with posterior CRP as described by Epley [64]. No mastoid vibration or any pre-medication was administered in our treatment procedures. Blood samples of 5 cc were drawn immediately after the CRP (BPPV-attacked samples). Patients were also instructed to keep their head upright for 2 hours and avoid lying down on the side of involvement. A follow up visit was arranged 1-2 weeks after the CRP (BPPV cured samples). A complete cure was defined as being symptom free and the absence of nystagmus in the Hallpike-Dix position. Blood samples (5 cc) were then drawn after the confirmation of a complete BPPV cure. All patients signed an informed consent form approved by Taipei Veterans General Hospital Institutional Review Board prior to the first blood sampling.

### Isolation of mRNA and quantitative real-time PCR

BPPV patient blood was collected in BD K2-EDTA tubes (5 ml) after maneuver intervention. The RNeasy Plus mini kit (Qiagen, Hilden, Germany) was used to isolate total blood RNA. The Experion Automated Electrophoresis Station (Bio-Rad) was used to check RNA quality. In this study, we used a homology search within the human genome (BLAST, National Center for Biotechnology Information, Bethesda, MD, USA) to determine oligonucleotide specificity, and the primers were confirmed by dissociation curve analysis. The oligonucleotide sequences are listed in Table 1. Oligonucleotides for SIRT1, DBC1, PGC-1a, p53, FoxO1, Foxo3, COX-2 and β-actin were designed using the computer software package Primer Express 2.0 (Applied Biosystems, Foster City, CA, USA). All oligonucleotides were synthesized by Invitrogen (Breda, The Netherlands). SYBR Green and an ABI 7000 sequence detection system (Applied Biosystems) were used to perform PCR according to the manufacturer's guidelines.

**Table 1:** The oligonucleotide sequences for real-time PCR assay

*Gene*	Sense	Anti-sense
**SIRT1**	5′-TGTGGTAGAGCTTGCATTGATCTT-3′	5′-GGCCTGTTGCTCTCCTCAT-3′
**DBC1**	5′-CCC TCG CCC GCC TAC TAT-3′	5′-GCT GGG CGG GGT TGT AGA-3′
**p53**	5′-GCCCACTTCACCGTACTAA-3′	5′-TGGTTTCAAGGCCAGATGT-3′
**PGC-1**	5′-CCGCACGCACCG AAA-3′	5′-TCGTGCTGATATTCCTCGTAGCT-3′
**PPAR-γ**	5′-AGTGTGAATTACAGC AAATCTCTGTTTT-3′	5′-GCACCATGCTCTGGGTCA A-3′
**FoxO1**	5′-ATGGTCAAGAGCGTGCCC-3′	5′-GATTGAGCATCCACCAAG-3′
**FoxO3**	5′-TCTCCCGTCAGCCAGTCTAT-3′	5′-AGTCACTGGGGA ACTTGTCG-3′
**β-actin**	5′-CGGGAAATCGTGCGTGAC-3′	5′-TGCCCAGGAAGGAAGGCT-3′
**miR-34a**	5′-CGGTATCATTTGGCAGTGTCT-3′	5′-GTGCAGGGTCCGAGGT-3′
**U6**	5′-GCCCGCTAGCTTATCAGACTGATG-3′	5′-GTGCAGGGTCCGAGGT-3′

### Investigation of microRNA expression levels

Blood miRNAs were isolated by PAXgene Blood miRNA Kit. Primers specific for miR-34a were obtained from Applied Biosystems (Foster City, CA, USA) to execute quantitative real-time PCR according to the vendor's protocol for TaqMan miRNA assays. Isolated total RNA from human plasma was initially processed for reverse transcription (RT) using two miRNA-specific primers (miR-34a: AB Assay ID 000426) to obtain RT products that were subsequently used for quantitative real-time PCR on a 7500 Fast System Real-Time PCR cycler (Applied Biosystems). U6 was used as a control to calculate the relative expression levels of miR-34a. Numerical indices of these expression levels are expressed using the 1/ΔCT method, and the values for each individual microRNA were obtained after subtracting the CT value of snoRNA202 (AB assay ID 001232, Applied Biosystems.

### ELISA assay for cytokines

After collection of human plasma from whole blood after 2500x g centrifuge at 4°C for 10 min. Human plasma was collected from whole blood after centrifugation at 2500 g at 4°C for 10 min. PEG2, IL-1beta, IL-6, IL-8 and TNF-α plasma concentrations were measured using an ELISA kit obtained from R&D Systems (Minneapolis, MN, USA). DBC1 plasma concentration was tested by as ELISA kit obtained from MyBioSource (San Diego, CA, USA)

### SOD activity measurement

After collection of human plasma from whole blood after 2500x g centrifuge at 4°C for 10 min. The SOD activity assay kit (Cell Biolabs, STA-340) was determined *via* an enzymatic assay method using a commercial kit according to the manufacturer's instructions. Enzymatic activity was revealed to units per milligram of protein.

### Hydrogen peroxide concentration assay

After collection of human plasma from whole blood after 2500x g centrifuge at 4°C for 10 min. The hydrogen peroxide assay kit (H_2_O_2_; Cell Biolabs, STA-344) was used to test the H_2_O_2_ concentration. The plasma concentration of hydrogen peroxide was shown to μM according to the manufacturer's instructions of the protocol.

### Statistical analyses

The data are expressed as the mean ± SD. The experimental results were analyzed using Paired sample *t*-test to compare hematological mRNA levels and plasma parameters. The significance level was set at *P* < 0.05.
